# Disruption of corporate financialization and labor cost growth: Evidence from China’s new asset management rules

**DOI:** 10.1371/journal.pone.0286683

**Published:** 2023-06-02

**Authors:** Chuyuan Liu

**Affiliations:** School of Accounting, Southwestern University of Finance and Economics, Chengdu, Sichuan, China; University of Malta, MALTA

## Abstract

The new asset management rules in China bring financial institutions under a unified regulatory framework, aiming to curb regulatory arbitrage, control systemic risk, and improve financial stability. Exploiting the new-rules-induced exogenous shock that disrupts corporate financialization, this study finds that firms with ex ante higher degrees of financialization respond to the regulation by increasing labor costs. Management’s financial expertise and ownership concentration are mechanisms through which disruption of financialization affects corporate employment strategies. The impact of the new rules on labor costs is more pronounced for firms with lower bankruptcy risk, located in coastal cities, and experiencing intense industry competition. The findings imply an unintended spillover effect of financial regulation on the labor market in the form of labor cost growth.

## 1. Introduction

In an attempt to reduce regulatory arbitrage and constrain systemic financial risk, China’s central government has undertaken several reforms to strengthen financial industry regulation. For example, the announcement in March 2023 to establish the National Financial Regulatory Administration suggests a realignment of responsibilities that requires organizational changes to unify regulatory functions and reduce regulatory arbitrage. A previous attempt to unify regulation took place in the asset management industry. The new asset management rules published in April 2018 are the first unified regulation to impose requirements for leverage ratios, risk reserve funds, and investment entry on all types of asset management products, which deters real sector firms from financializing. Research discusses how the new rules affect corporate investment [1]. Little attention has been paid to how the unified regulation in the asset management market shapes non-financial firms’ employment behavior. This paper focuses on the new asset management rules aimed at curbing regulatory arbitrage and investigates how firms in non-financial sectors adjust their employment decisions in response to the disruption of corporate financialization.

Despite investments of $16.1 trillion by the end of 2016, China’s asset management market lacks the unified regulatory framework, resulting in excessive leverage and risks from mismatches. The new asset management rules rolled out in April 2018 forbid financial institutions from offering investors guaranteed returns and place tighter oversight on asset management products and services. A key objective of the new rules is to promote the development of real economy, and non-financial sector firms have fewer opportunities for financial investments and are discouraged from engaging in corporate financialization. In other words, the new rules are an exogenous shock that disrupts the process of financialization for real sector firms.

The rate of return gap between financial investments and real investments motivates firms to engage in financialization [2]. A strand of literature provides evidence that these financial profit opportunities would crowd out real investments [3–6]. Since regulatory arbitrage of liquidity rules contributes to the rapid growth of China’s shadow banking sector [7], the first unified rules in the asset management market impose stricter supervision on financial innovation products, constraining real sector firms’ involvement in financialization. The disruption of corporate financialization prompts firms to refocus on real-economy activities, and firms with ex ante higher levels of financialization are in greater need of an overhaul to regain competitiveness. Firms’ human capital plays a crucial role in its competitiveness and success [8, 9]. Considering that attracting employees requires competitive compensations [10–13], this study therefore expects that firms with ex ante higher degrees of financialization respond to the issuance of the new rules by increasing labor costs.

To examine the relationship between corporate financialization and firms’ employment decisions, this paper exploits the new asset management rules as an exogenous shock to firms’ involvement in financialization and conducts the difference-in-differences estimation. Specifically, firms with ex ante higher degrees of financialization are identified by the ratio of financial investments to total assets one year prior to the launch of the new rules [2, 6]. The pre-treatment period is 2014–2017, and the post-treatment period is 2018–2021. For dependent variables, the percentage changes in both cash expenses and total compensation paid to rank-and-file employees are investigated. The findings suggest that real sector firms with ex ante higher levels of financialization respond to the new rules by larger growth in labor costs. The parallel trend analysis indicates that there is no pre-existing trend in labor costs before the launch of the new rules. The baseline results are also robust to a battery of tests, including placebo tests, propensity score matching, and alternative specifications.

Next, the research examines the underlying mechanisms through which non-financial firms respond to the financialization disruption by different levels of adjustments in employment strategies. The rate of return gap between financial and real sector activities drives managers to favor short-term financial investments over long-term real investments, which facilitates meeting short-term earnings goals and pursuing private benefits. Besides, managers with financial expertise are adept at spotting opportunities for financial profits. Furthermore, ownership concentration makes it convenient for the largest shareholder to shift financial profits. In light of this, agency costs arising from managerial short-termism, management’s pursuit of private benefits, and ownership concentration could contribute to firms’ lingering on financialization and reluctance to refocus on real sector activities. The results indicate that highly financialized firms with less financially savvy management and less severe ownership concentration are more agile in increasing labor costs as a response to the new rules.

Moreover, the paper investigates the heterogeneity in the impact of the new rules on corporate labor costs. Firms exposed to higher bankruptcy risk are financially distressed and have limited resources to execute new corporate strategies. Thus, the impact of the new rules on corporate labor costs would be attenuated for firms in financial distress. Coastal regions have faster economic growth, a more welcoming business environment, and better health and education resources, resulting in more talent inflows [14–17]. When seeking real-economy opportunities, firms located in coastal cities are more likely to hire talent appropriate for the task at hand. Hence, the effect of financialization disruption on corporate labor costs would be greater for firms located in coastal cities. Employee engagement contributes to firms’ competitiveness and value, and could be enhanced through better employee treatment [18, 19], especially for firms in competitive industries. Pecuniary inputs for improving employee treatment are more likely to occur when firms operate in industries with heavy competition, and therefore the impact of the new rules on corporate labor costs would be stronger for these firms. Consistent with the expectation, the effects of the new-rules-induced financialization disruption on corporate labor costs are more pronounced for firms exposed to lower bankruptcy risk, located in coastal cities, and competing in highly competitive industries.

This study contributes to the literature on regulatory arbitrage by examining the externality of regulation in the asset management market. Strict regulation on banks gives rise to their regulatory arbitrage motivation, resulting in banks’ riskier activities and the rapid growth in shadow banking [20–23]. The new rules target financial institutions’ regulatory arbitrage and put them under a clearer regulatory framework, which disrupts real sector firms’ financialization and affects their employment decisions. The results imply that financial regulations aimed at constraining regulatory arbitrage could have an unintended and significant spillover effect in the labor market through increasing labor costs.

In addition, this study contributes to the literature on corporate financialization by exploiting an exogenous shock to the asset management market. Prior research focuses on the motivations and consequences of non-financial firms’ financialization, suggesting that financial investments crowd out real investments [2, 4–6]. A few studies examine the motivations and capital market consequences of de-financialization policies [1, 24]. By exploiting an exogenous shock that disrupts corporate financialization, this paper provides direct empirical evidence of how the de-financialization policy impacts the labor market through adjustment in corporate employment decisions.

As a regulation aimed to control systemic financial risk and improve financial stability, the new asset management rules are valued by policymakers due to the unified regulatory framework. This paper contributes to financial regulation in the Chinese context by providing empirical evidence that unifying financial regulation functionally could have real effects on the behavior of microeconomic agents.

The remainder of the paper proceeds as follows. Section 2 discusses the institutional background and develops the hypothesis. Section 3 explains the research design. Section 4 presents the main empirical results and Section 5 discusses robustness tests and further analysis. Section 6 presents discussion and limitations of the study, followed by conclusions, implications, and future research directions in Section 7.

## 2. Literature review and hypothesis development

### 2.1 Literature review on corporate financialization

A classic topic in the economics literature discusses the optimal allocation of resources for portfolios. Tobin [25] provides insights that the allocation of resources between real and financial investments hinges on their respective rates of return. Corporate financialization diverts resources away from the real economy and a strand of literature focuses on corporate financialization and examines the substitution between real and financial assets existing in real sector firms in developed countries [3–5]. Demir [2] investigates financial liberalization in three emerging markets and finds that real sector firms choose short-term reversible financial investments over long-term irreversible fixed investments because of the rate of return gap and uncertainty in the economy. Though some studies suggest the bright side of corporate financialization [26, 27], financialization in Chinese real sector firms could serve as a speculative tool, resulting in opaque financial reports and stock price crashes [28, 29].

Despite ample evidence of the role that corporate financialization plays in the capital market [2–6, 30], little is known about how de-financialization affects firms’ employment decisions. A few papers investigate the effect of de-financialization on corporate investments and operating risks in the Chinese context [1, 31]. Gonzalez and Sala [32] conduct simulations of macroeconomic conditions in the United States and find the relationship between financialization and unemployment in a developed country. Yet, the scarcity of direct evidence still exists that corporate financialization interacts with the labor market in an emerging economy.

### 2.2 The new asset management rules as the exogenous shock to corporate financialization

In order to tame the risks in the financial system, the new asset management rules, published by China’s top policymakers in April 2018, bring the asset management industry under a unified regulatory framework. All types of asset management products are subject to the new rules, which restrict investment entry and prohibit financial institutions from offering investors guaranteed returns. Before penalties are imposed, financial institutions are required to adjust their relevant business during the grace period of one year. Anecdotal evidence shows that the size of shadow banking plummeted by 13% after the release of the new rules and that newly registered asset management products also dropped by 55% in early 2018 [33].

With the publication of the new rules, the asset management industry has undergone a major overhaul, limiting the opportunities for firms in non-financial sectors to invest in financial assets and narrowing down the rate of return gap between financial investments and real-economy investments. The shrunk access to and pecuniary motivation for financial investments would quench the enthusiasm for financialization among real sector firms. Thus, the new asset management rules are seen as an exogenous shock to the pursuit of financialization for firms in non-financial sectors.

### 2.3 Hypothesis development

Human capital is crucial for firms’ development and competitiveness [8, 9]. To retain and attract human capital, firms are supposed to offer competitive salaries and non-financial employee benefits [10–13, 34]. With the discretion to adjust corporate policies, market-oriented Chinese firms are able to adjust their labor hiring decisions based on market conditions and corporate strategies [35, 36]. The new rules tighten oversight of the massive asset management market and represent an exogenous shock to firms’ financialization process, pressuring firms in non-financial sectors to de-financialize and rethink their strategies concerning real sector activities. Facing the regulation-induced changes in market conditions, these firms would have to adjust their labor hiring strategies and experience a variation in labor costs.

According to agency theory, firms could participate in financialization as managers pursue private benefits. The literature indicates that the rate of return gap between financial and real investments offers managers opportunities for speculation [2], especially when managers hold company stock or receive option-based compensation. Furthermore, agency conflicts between minority shareholders and controlling shareholders could also lead firms to engage in financialization. Ownership concentration is prevalent in China and gives rise to expropriation [37], enabling controlling shareholders to profit from financial investments. Aggravated agency conflicts prompt managers and controlling shareholders of real sector firms to divert resources to financial investments and engage in financialization out of speculation, crowding out the investments in the real economy [6, 25, 38].

The publication of the new asset management rules urges financial institutions to reshape products and services, diminishing the access to and the excess return on financial investments for real sector firms. In light of this, the substitution of financial investments for real investments is curbed and non-financial firms would divert resources back to real activities. The changes in corporate strategies require reorganization of business and adjustments to employment decisions, and firms with ex ante higher degrees of financialization would make a sharper redirection. I expect that real sector firms respond to the exogenous shock by increasing labor costs to retain and attract human capital.

However, possibilities exist that financialized firms would reduce labor costs after the launch of the new rules. Based on the resource dependency theory, non-financial firms’ financialization could reduce reliance on external financing by supplementing credit resources with returns on financial investments [27, 39]. Furthermore, firms could utilize returns on financial investments to improve balance sheets and lower financing costs when investing in real-economy activities [40]. According to this, stricter restrictions on asset management products constrain the resources for real-economy investments, resulting in firms downsizing rank-and-file employees to prepare for potential financial distress. Thus, firms with ex ante higher levels of financialization are more likely to streamline their labor force and reduce labor costs in response to the exogenous shock imposed by the new rules.

The latter scenario is of less likelihood for the following reasons. First, the new rules are the first unified regulatory framework aimed at curbing regulatory arbitrage and controlling systemic financial risks, putting the asset management industry under more rigorous oversight than ever before. The investment entry and the cooling down on financial product innovations restrict real sector firms from engaging in financial investments and increase their real investments [1], prompting firms to hire more labor force. With them increasing the need for labor, the non-financial firms’ competition in the labor market further increases the cost of attracting and retaining human capital. I therefore argue that the former scenario would dominate the research context and present the hypothesis as follows:

H1. Firms with ex ante higher degrees of financialization respond to the new rules by a larger increase in labor costs.

## 3. Research design

### 3.1 Data and sample

Initially, the sample data covers all publicly listed companies on the Shanghai Stock Exchange (SHSE) and the Shenzhen Stock Exchange (SZSE) from 2014 to 2021. Since the new asset management rules were published in April 2018, I set the post treatment period as 2018–2021. Firms in the financial industry are excluded from the sample due to their special reporting requirements [41]. I also exclude firms without positive total assets and those with missing values on key variables. The data are primarily collected from the CNRDS (Chinese Research Data Services Platform) database and supplemented by the CSMAR (China Stock Market and Accounting Research) database. The final sample consists of 23,552 firm-year observations.

### 3.2 Empirical strategy

Using the setting of the new asset management rules, I adopt the difference-in-differences approach to test the hypothesis. Since the new rules do not impact on firms’ employment decisions directly, the effect on labor costs is likely exogenous. The equation for the baseline analyses is as follows:

$$LaborCost_{i,t}=\;\alpha +\;\beta CorpFin_{i}\times Post_{t}+\;\gamma_{k}Controls++Fixed\;Effects+\;\varepsilon_{i,t}$$ (1)

where *i* and *t* denote the firm and year, respectively. Following Bertrand et al. [42], I focus on firms’ degrees of corporate financialization one year prior to the launch of the new rules [41] and construct the variable *CorpFin* to measure the extent to which firms are exposed to the new rules. Based on Orhangazi [6] and Demir [2], *CorpFin* is defined as the ratio of the firm’s financial assets to its total assets. Following Du et al. [43], financial assets include transactional financial assets, derivative financial assets, net values of loans and advances, net values of available-for-sale financial assets, net values of investments held to maturity, and net values of real estate for investing. *Post* is a dummy variable equal to one for years 2014–2017, and zero for years 2018–2021. I employ two variables, *LaborCost_Cash* and *LaborCost_Total*, to measure the percentage changes in firms’ employment decisions [44, 45]. Specifically, *LaborCost_Cash* is defined as the percentage change in cash expenses paid to employees, where employee cash expenses are equal to the “cash paid to employees” in the cash flow statement minus total executive compensation. *LaborCost_Total* is defined as the percentage change in total expenses paid to employees, where employee total expenses are the sum of employee cash expenses and the change in the "employee compensation payable" in the balance sheet.

Based on prior literature [13, 46–48], firm-level control variables include size (*Size*), leverage (*Lev*), age (*Age*), capital intensity (*PPE*), operating cash flows (*OpeCashFlow*), return on assets (*ROA*), and sales growth (*SalesGrowth*). Following Liu et al. [41], province-level determinants of labor costs are also considered in Eq (1), including GDP per capita (*ProvGDP*), GDP growth (*ProvGDPGrowth*), population (*ProvPop*), and wage per capita (*ProvWage*). The definitions and data sources for all variables are presented in Table 1.

**Table 1:** Variable definitions.

Variable	Definition
LaborCost_Cash	The percentage change in cash expenses paid to employees; where cash expenses paid to employees are equal to the “cash paid to employees” in the cash flow statement minus total executive compensation. (CNRDS)
LaborCost_Total	The percentage change in total expenses paid to employees; where total expenses paid to employees are equal to the “cash paid to employees” in the cash flow statement plus the change in the "employee compensation payable" in the balance sheet, minus total executive compensation. (CNRDS)
CorpFin	The degree of corporate financialization one year prior to the new asset management rules’ launch. Based on Orhangazi [6] and Demir [2], a firm’s corporate financialization degree is defined as the ratio of the firm’s financial assets to its total assets. Following Du et al. [43], financial assets include transactional financial assets, derivative financial assets, net values of loans and advances, net values of available-for-sale financial assets, net values of investments held to maturity, and net values of real estate for investing. (CNRDS)
Post	A dummy variable set equal to 1 for years 2014–2017, and 0 for years 2018–2021. (CNRDS)
Size	The natural logarithm of total assets. (CNRDS)
Lev	Total liabilities scaled by total assets. (CNRDS)
Age	The natural logarithm of firm age. (CNRDS)
PPE	The natural logarithm of property, plant, and equipment. (CNRDS)
OpeCashFlow	Operating cash flow divided by total assets. (CNRDS)
ROA	Return on assets (in percentage). (CNRDS)
SalesGrowth	The percentage change in sales revenue from the previous year to the focal year. (CNRDS)
ProvGDP	The natural logarithm of GDP per capita of the province. (CSMAR)
ProvGDPGrowth	The percentage change in GDP of the province from the previous year to the focal year. (CSMAR)
ProvPop	The natural logarithm of population of the province. (CSMAR)
ProvWage	The natural logarithm of wage per capita of the province. (CSMAR)
Manage_Fin	An indicator variable that equals 1 if the management has work experience in the financial industry, and 0 otherwise. (CSMAR)
HighConcentration	An indicator variable that equals 1 if Concentration of the firm is above the median, and 0 otherwise. Concentration is defined as the ratio of the shares held by the largest shareholder to the shares held by the second largest shareholder. (CNRDS)
HighBankruptcyRisk	An indicator variable that equals 1 if the firm’s bankruptcy risk is above the median in year t-1, and 0 otherwise. Firms’ bankruptcy risks are computed as in Altman [55]. (CSMAR)
CoastalCity	An indicator variable that equals 1 if the firm is located in the coastal city, and 0 otherwise. (CNRDS)
HighCompetition	An indicator variable that equals 1 if the firm is in the industry with above-median *Competition*, and 0 otherwise. *Competition* is the Herfindahl-Hirschman Index based on sales revenue. (CSMAR)
PseudoPost	A dummy variable set equal to 1 for years 2014–2015, and 0 for years equal to or larger than 2016. (CNRDS)
CorpFin_2Yr	The average degree of corporate financialization two years prior to the new rules’ launch. Based on Orhangazi [6] and Demir [2], a firm’s corporate financialization degree is defined as the ratio of the firm’s financial assets to its total assets. (CNRDS)
CorpFin_PreTreat	The average degree of corporate financialization during the pre-treatment period before the new rules’ launch. Based on Orhangazi [6] and Demir [2], a firm’s corporate financialization degree is defined as the ratio of the firm’s financial assets to its total assets. (CNRDS)
LaborN	The change in the number of rank-and-file employees. (CSMAR)

The regression model includes firm fixed effects to control for time-invariant heterogeneity across firms and year fixed effects to control for time-specific effects that could affect firms’ employment decisions. Robust standard errors are clustered at the firm level and all continuous variables are winsorized at the 1% and 99% levels.

### 3.3 Descriptive statistics

Descriptive statistics for the key variables are presented in Table 2. The median, mean, and standard deviation values of *CorpFin* are 0.0122, 0.0418, and 0.0803, respectively, suggesting that a relatively smaller proportion of the sample firms were deeply involved in corporate financialization before the new rules were published. The mean and standard deviation values of *LaborCost_Cash* are 16.31% and 32.11%, respectively, indicating that the percentage change in cash paid to rank-and-file employees obviously differs. The statistics of *LaborCost_Total* show similar results. The summary statistics for the control variables are similar to prior literature [36].

**Table 2:** Descriptive statistics.

	Obs	Mean	Median	SD	Min	Max
LaborCost_Cash	23552	16.3104	10.9968	32.1069	-45.7093	195.6350
LaborCost_Total	22409	16.4175	10.3370	36.1287	-49.5177	239.2427
CorpFin	23552	0.0418	0.0122	0.0803	-0.0002	0.9534
Post	23552	0.5531	1.0000	0.4972	0.0000	1.0000
Size	23552	22.3107	22.1509	1.3068	19.7028	26.2296
Lev	23552	0.4398	0.4284	0.2091	0.0580	0.9624
Age	23552	2.9625	2.9957	0.2865	2.0794	3.5835
PPE	23552	20.2684	20.2417	1.7588	15.0471	24.6816
OpeCashFlow	23552	0.0456	0.0450	0.0695	-0.1745	0.2425
ROA	23552	2.6219	3.2700	8.2326	-41.4300	20.0500
SalesGrowth	23552	17.4543	9.7884	48.9412	-65.7433	327.3001
ProvGDP	23552	11.2793	11.3129	0.4189	10.3525	12.1226
ProvGDPGrowth	23552	6.7453	7.1534	2.0164	0.6000	10.7994
ProvPop	23552	8.5672	8.6742	0.6444	6.5805	9.4481
ProvWage	23552	11.3548	11.3122	0.3278	10.7386	12.1790

Notes: This table reports summary statistics. The data on labor costs and firm fundamentals are obtained from the CNRDS database, and the province-level data are sourced from the CSMAR database.

## 4. Empirical results

### 4.1 Baseline results

To analyze how firms with larger exposure to the new rules respond by adjusting employment decisions, I regress labor cost growth on the interaction term of firms’ degrees of financialization and the post-event indicator (*CorpFin×Post*). The results are reported in Table 3. In column (1) with *LaborCost_Cash* as the dependent variable, the estimated coefficient of *CorpFin×Post* is positive and significant at the 1% level, suggesting that firms respond to the disruption of corporate financialization by increasing cash expenses paid to rank-and-file employees. The estimate of *CorpFin×Post* for *LaborCost_Total* in column (2) is significantly positive at the 5% level. The effects are also economically significant. For example, following the launch of the new rules, a one-standard-deviation increase in *CorpFin* is associated with a 7.66% increase in firms’ cash wage expenses relative to their mean value.

**Table 3:** The disruption of corporate financialization and labor costs.

	LaborCost_Cash	LaborCost_Total
	(1)	(2)
CorpFin×Post	15.5628***	13.4318**
	(2.80)	(2.22)
Size	9.1600***	9.1735***
	(10.53)	(9.38)
Lev	-4.4920	-7.4201**
	(-1.59)	(-2.33)
Age	-11.1714**	-8.0582
	(-2.14)	(-1.45)
PPE	0.6341	0.6320
	(1.03)	(0.87)
OpeCashFlow	-13.4896***	-3.9928
	(-3.05)	(-0.80)
ROA	0.0596	-0.0908*
	(1.44)	(-1.96)
SalesGrowth	0.3381***	0.4017***
	(32.12)	(31.49)
ProvGDP	-1.8388	-2.8270
	(-0.69)	(-0.91)
ProvGDPGrowth	0.0039	0.1568
	(0.02)	(0.54)
ProvPop	5.0999	6.4331
	(1.28)	(1.37)
ProvWage	-2.3086	-0.5413
	(-0.42)	(-0.08)
Constant	-168.3761**	-199.2908**
	(-2.45)	(-2.53)
*Firm fixed effects*	YES	YES
*Year fixed effects*	YES	YES
*N*	23552	22406
Adj. *R*^2^	0.387	0.379

Notes: This table examines the effect of financialization disruption on labor costs. The data on labor costs and firm fundamentals are obtained from the CNRDS database, and the province-level data are sourced from the CSMAR database. *LaborCost_Cash* is the percentage change in cash expenses paid to employees, and *LaborCost_Total* is the percentage change in total expenses paid to employees. *CorpFin* is the ratio of the firm’s financial assets to its total assets one year prior to the new rules’ launch. *Post* is a dummy variable equal to 1 for years 2014–2017, and 0 for years 2018–2021. All variables are defined in the Table 1. Robust standard errors are clustered at the firm level and *t*-statistics are reported in parentheses.

***, **, and * indicate statistical significance at the 1%, 5%, and 10% levels, respectively.

### 4.2 Parallel trend analysis

To establish the causal link between the new asset management rules and corporate employment decisions, the parallel trend assumption of the difference-in-differences estimation should be validated. Specifically, the new rules are proved as an exogenous shock when the changes in labor costs coincide with the disruption of corporate financialization. Following Bertrand and Mullainathan [49], I replace the variable *Post* with year indicator variables before or after the publication of the new rules. For example, *Before3* is a dummy variable indicating three years before the launch year of the new rules, and *After2+* is an indicator variable representing at least two years after the launch year.

Table 4 presents the results. The estimated coefficients of *CorpFin×Before3*, *CorpFin×Before2*, and *CorpFin×Before1* are insignificant, suggesting that there were no pre-existing changes in labor costs for firms with different levels of financialization prior to the publication of the new rules. Moreover, the coefficients of *CorpFin×After0*, *CorpFin×After1*, and *CorpFin×After2+* are significantly positive. The precise timing of the changes in labor costs indicates that the new rules are an exogenous shock and illustrates how the treatment effects evolve over time.

**Table 4:** Parallel trend analysis.

	LaborCost_Cash	LaborCost_Total
	(1)	(2)
CorpFin×Before3	3.3627	12.7431
	(0.33)	(0.95)
CorpFin×Before2	9.3046	14.5644
	(0.97)	(1.28)
CorpFin×Before1	6.7254	6.3027
	(0.62)	(0.50)
CorpFin×After0	18.4587*	20.8568*
	(1.82)	(1.78)
CorpFin×After1	28.2293**	31.4993**
	(2.17)	(2.29)
CorpFin×After2+	17.6590*	17.3485*
	(1.84)	(1.65)
Size	9.1504***	9.1584***
	(10.50)	(9.36)
Lev	-4.4676	-7.3939**
	(-1.58)	(-2.32)
Age	-11.1442**	-8.1761
	(-2.13)	(-1.46)
PPE	0.6440	0.6440
	(1.04)	(0.88)
OpeCashFlow	-13.5220***	-4.0883
	(-3.06)	(-0.82)
ROA	0.0596	-0.0911*
	(1.44)	(-1.96)
SalesGrowth	0.3381***	0.4017***
	(32.10)	(31.48)
ProvGDP	-2.0115	-2.9917
	(-0.75)	(-0.97)
ProvGDPGrowth	-0.0041	0.1468
	(-0.02)	(0.50)
ProvPop	5.1253	6.4858
	(1.29)	(1.38)
ProvWage	-2.0139	-0.1668
	(-0.36)	(-0.03)
Constant	-170.2269**	-201.9952**
	(-2.48)	(-2.57)
*Firm fixed effects*	YES	YES
*Year fixed effects*	YES	YES
*N*	23552	22406
Adj. *R*^2^	0.387	0.379

Notes: This table conducts the parallel trend analysis. The data on labor costs and firm fundamentals are obtained from the CNRDS database, and the province-level data are sourced from the CSMAR database. *Before3*, *Before2*, *Before1*, *After0*, *After1*, and *After2+* are year indicator variables before or after the launch of the new rules. All variables are defined in the Table 1. Robust standard errors are clustered at the firm level and *t*-statistics are reported in parentheses.

***, **, and * indicate statistical significance at the 1%, 5%, and 10% levels, respectively.

### 4.3 Underlying mechanisms

In section 2, I argue that agency costs play an essential role in financialization for real sector firms, and that the induced disruption of financialization facilitates firms’ redirection to the real-economy activities, thereby increasing labor costs. To provide more direct evidence that agency costs affect firms’ de-financialization process, I conduct firm-level analyses to investigate how agency costs interact with the impact of the new rules on labor costs. The regression model has the following general form:

$$\begin{array}{ll} LaborCost_{i,t} & =\;\alpha +\;\beta_{1}CorpFin_{i}\times Post_{t}\times Agency_{i,t}+\beta_{2}CorpFin_{i}\times Post_{t}+\beta_{3}CorpFin_{i}\times Agency_{i,t}+\\ & \beta_{4}Post_{t}\times Agency_{i,t}+\beta_{5}Agency_{i,t}+\;\gamma_{k}Controls++Fixed\;Effects+\;\varepsilon_{i,t} \end{array}$$ (2)

*Agency* representing the variables, *Manager_Fin* and *HighConcentration*, that proxy for agency costs from different dimensions, which will be discussed in detail later. The model includes firm fixed effects and year fixed effects, and other details of specification remain the same as Eq (1).

#### 4.3.1 Management with financial expertise

Management’s decision-making is heavily influenced by career concerns and pursuit of private benefits [50, 51]. Since the rate of return gap between financial and real investments drives corporate financialization [2], management’s pressure of boosting stock prices and maximizing shareholder value increases the likelihood of managerial short-termism, leading them to seek out financial investments that yield rapid returns [4–6, 52]. Accordingly, compared to long-term irreversible real investments, short-term reversible financial investments are more likely to fulfill managers’ career goals and pursuit of private benefits. Though the new rules impose strict oversight on financial innovation products and investment entry, the management with financial expertise is shrewd in circumventing limitations and discovering opportunities for financial investments. In other words, the management with financial expertise has motivations and abilities to linger on the strategy of corporate financialization after the launch of the new rules. Hence, I expect that highly financialized firms with financially savvy management are less likely to increase labor costs in response to the new rules.

Specifically, I employ the variable, *Manage_Fin*, to proxy for managerial agency costs. *Manage_Fin* is an indicator variable that equals one if the management has work experience in the financial industry, and zero otherwise. The results are presented in Table 5. The estimated coefficients of *CorpFin×Post×Manage_Fin* in columns (1) and (2) are negative and significant at the 10% level, suggesting that ex ante highly financialized firms with financially sophisticated management are more likely to suppress labor cost growth in response to the disruption of financialization. The results imply that real sector firms’ redirection to the real economy may be sluggish due to managerial agency costs.

**Table 5:** Mechanism: Management with financial expertise.

	LaborCost_Cash	LaborCost_Total
	(1)	(2)
CorpFin×Post×Manage_Fin	-21.3251*	-27.8172*
	(-1.70)	(-1.74)
CorpFin×Post	34.7838***	38.0553**
	(2.92)	(2.56)
CorpFin×Manage_Fin	-9.0577	-8.8289
	(-1.10)	(-0.84)
Post×Manage_Fin	-1.3231	-1.3276
	(-1.52)	(-1.31)
Manage_Fin	0.2437	0.2356
	(0.33)	(0.26)
Size	9.2327***	9.2618***
	(10.59)	(9.44)
Lev	-4.5685	-7.4978**
	(-1.61)	(-2.35)
Age	-11.3783**	-8.2936
	(-2.18)	(-1.49)
PPE	0.5771	0.5718
	(0.94)	(0.78)
OpeCashFlow	-13.4210***	-3.8926
	(-3.04)	(-0.78)
ROA	0.0571	-0.0938**
	(1.38)	(-2.02)
SalesGrowth	0.3378***	0.4013***
	(32.13)	(31.49)
ProvGDP	-1.8459	-2.8867
	(-0.69)	(-0.93)
ProvGDPGrowth	0.0029	0.1572
	(0.01)	(0.54)
ProvPop	5.3163	6.6980
	(1.34)	(1.43)
ProvWage	-2.2752	-0.4227
	(-0.41)	(-0.07)
Constant	-169.7366**	-201.6404**
	(-2.47)	(-2.57)
*Firm fixed effects*	YES	YES
*Year fixed effects*	YES	YES
*N*	23552	22406
Adj. *R*^2^	0.387	0.379

Notes: This table investigate the mechanism through which financialization disruption affects labor cost growth. The data on labor costs and firm fundamentals are obtained from the CNRDS database, and the data on provinces and management’s financial expertise are sourced from the CSMAR database. *Manage_Fin* is an indicator variable that equals 1 if the management has work experience in the financial industry, and 0 otherwise. All variables are defined in the Table 1. Robust standard errors are clustered at the firm level and *t*-statistics are reported in parentheses.

***, **, and * indicate statistical significance at the 1%, 5%, and 10% levels, respectively.

#### 4.3.2 Ownership concentration

Ownership concentration is prevalent in China [53], and weak investor protection exacerbates agency costs between controlling and minority shareholders [54]. Controlling shareholders are equipped with multiple tools to expropriate from minority shareholders [37], which makes it more feasible and appealing to reap gains from financial investments. In light of this, firms with ownership concentration are more likely to adopt a wait-and-see attitude towards the unified rules imposed in the asset management market, resulting in a slim possibility of labor cost growth.

To empirically examine the interaction between ownership concentration and the adjustment to labor costs after the new rules’ launch, I use the variable *HighConcentration* to identify firms with higher levels of ownership concentration. *HighConcentration* is an indicator variable that equals one if *Concentration* of the firm is above the median, and zero otherwise. *Concentration* is defined as the ratio of the shares held by the largest shareholder to the shares held by the second largest shareholder. The results are shown in Table 6. The estimated coefficients of *CorpFin×Post×HighConcentration* are significantly negative at the 5% level with *LaborCost_Cash* and *LaborCost_Total* as the dependent variables, respectively, suggesting that agency costs induced by ownership concentration would suppress highly financialized firms’ adjustment to labor costs as the response to the new rules.

**Table 6:** Mechanism: Ownership concentration.

	LaborCost_Cash	LaborCost_Total
	(1)	(2)
CorpFin×Post×HighConcentration	-24.4977**	-26.3555**
	(-2.12)	(-2.12)
CorpFin×Post	28.1979***	26.8598***
	(3.39)	(2.98)
CorpFin×HighConcentration	-14.3285	-19.2546
	(-1.23)	(-1.40)
Post×HighConcentration	1.9993**	3.0637***
	(2.24)	(3.00)
HighConcentration	-0.1182	-0.0401
	(-0.13)	(-0.04)
Size	9.1648***	9.1855***
	(10.56)	(9.43)
Lev	-4.4595	-7.3471**
	(-1.57)	(-2.30)
Age	-11.3486**	-8.2100
	(-2.18)	(-1.47)
PPE	0.6008	0.5958
	(0.99)	(0.83)
OpeCashFlow	-13.3638***	-3.8128
	(-3.02)	(-0.76)
ROA	0.0582	-0.0942**
	(1.41)	(-2.03)
SalesGrowth	0.3379***	0.4015***
	(32.08)	(31.46)
ProvGDP	-1.9716	-3.1225
	(-0.74)	(-1.01)
ProvGDPGrowth	-0.0092	0.1426
	(-0.04)	(0.49)
ProvPop	5.2533	6.5989
	(1.33)	(1.41)
ProvWage	-2.1143	-0.1462
	(-0.38)	(-0.02)
Constant	-169.4150**	-201.2991**
	(-2.47)	(-2.57)
*Firm fixed effects*	YES	YES
*Year fixed effects*	YES	YES
*N*	23550	22404
Adj. *R*^2^	0.387	0.379

Notes: This table investigate the mechanism through which financialization disruption affects labor cost growth. The data on labor costs and firm fundamentals are obtained from the CNRDS database, and the province-level data are sourced from the CSMAR database. *HighConcentration* is an indicator variable that equals 1 if ownership concentration of the firm is above the median, and 0 otherwise. All variables are defined in the Table 1. Robust standard errors are clustered at the firm level and *t*-statistics are reported in parentheses.

***, **, and * indicate statistical significance at the 1%, 5%, and 10% levels, respectively.

### 4.4 Heterogeneity across firms, regions, and industries

In this section, I explore the heterogeneity of treatment effects by conducting subsample tests. Specifically, three perspectives are analyzed: firms’ bankruptcy risk, regional economic development, and industry competition.

#### 4.4.1 Bankruptcy risk

The baseline results show that firms with ex ante higher degrees of financialization respond to the new rules by increasing more labor costs. The refocus on human capital requires adjustments to corporate strategy and inputs of resources. After the publication of the new rules, financially distressed firms are less likely to improve employee treatment because of higher bankruptcy risk and therefore the widened gap between available and adjustment-required resources. Thus, I expect that the impact of the new rules on labor cost growth is attenuated for highly financialized firms with higher bankruptcy risk.

To test this conjecture, I divide the full sample into high and low bankruptcy risk groups based on the median of *HighBankruptcyRisk* and then re-estimate Eq (1). A firm’s bankruptcy risk is measured as the Altman’s Z-score in year *t-1* [55]. The results are reported in Table 7. The estimated coefficients of *CorpFin×Post* are significantly positive in the low bankruptcy risk group while become insignificant for the group with high bankruptcy risk, suggesting that the impact of the new rules on labor cost growth is attenuated for highly financialized firms with greater likelihood of bankruptcy.

**Table 7:** Cross-sectional variation: Bankruptcy risk.

	LaborCost_Cash		LaborCost_Total	
	High Bankruptcy Risk	Low Bankruptcy Risk	High Bankruptcy Risk	Low Bankruptcy Risk
	(1)	(2)	(3)	(4)
CorpFin×Post	-2.4665	20.2480**	-4.9299	23.4736**
	(-0.41)	(2.19)	(-0.68)	(2.23)
Size	8.7445***	12.8026***	9.6701***	13.0102***
	(6.78)	(8.23)	(6.66)	(6.63)
Lev	-11.8699***	17.5026***	-12.5739***	18.3851***
	(-2.90)	(3.40)	(-2.89)	(2.93)
Age	4.0982	-34.6267***	7.1716	-26.1292***
	(0.57)	(-4.10)	(0.92)	(-2.82)
PPE	1.5108*	0.3295	1.3717	0.9848
	(1.79)	(0.33)	(1.21)	(0.78)
OpeCashFlow	-21.9816***	-4.5797	-17.4931***	9.2778
	(-3.67)	(-0.69)	(-2.64)	(1.25)
ROA	-0.0147	-0.0317	-0.1572***	-0.1975**
	(-0.27)	(-0.48)	(-2.71)	(-2.56)
SalesGrowth	0.3455***	0.3206***	0.3856***	0.4007***
	(22.04)	(19.50)	(20.48)	(20.24)
ProvGDP	-2.1995	-4.3310	-1.6020	-4.8719
	(-0.56)	(-1.11)	(-0.36)	(-1.07)
ProvGDPGrowth	0.2917	-0.1224	0.4420	-0.0789
	(0.94)	(-0.34)	(1.33)	(-0.17)
ProvPop	5.9081	-6.8314*	7.3184	-7.1694
	(1.06)	(-1.65)	(1.11)	(-1.36)
ProvWage	-1.3013	-5.8833	-5.3692	-7.5452
	(-0.17)	(-0.78)	(-0.62)	(-0.84)
Constant	-240.9250***	-0.9564	-242.9464**	-17.7171
	(-2.58)	(-0.01)	(-2.43)	(-0.13)
*Firm fixed effects*	YES	YES	YES	YES
*Year fixed effects*	YES	YES	YES	YES
*Empirical p-value*	0.000		0.000	
*N*	11069	10976	10667	10229
Adj. *R*^2^	0.383	0.428	0.364	0.440

Notes: This table investigates the heterogeneity in the baseline effects across firms. The data on labor costs and firm fundamentals are obtained from the CNRDS database, and the data on provinces and corporate bankruptcy risk are sourced from the CSMAR database. *HighBankruptcyRisk* is an indicator variable that equals 1 if the firm’s Z-score is above the median in year t-1, and 0 otherwise. All variables are defined in the Table 1. Robust standard errors are clustered at the firm level and *t*-statistics are reported in parentheses.

***, **, and * indicate statistical significance at the 1%, 5%, and 10% levels, respectively.

#### 4.4.2 Coastal cities

Coastal regions in China enjoy favorable economic policies and benefit from the coastal development strategy [56, 57]. Besides, improvements in the legal environment and less government intervention promote faster growth in the market economy [15, 16], fostering a friendly environment for business. Furthermore, advantages in income, health, and education draw people to coastal regions [14], making it easier for real sector firms to recruit talent for development. Thus, I expect that the effect of the new rules on labor cost growth is more pronounced for those firms located in coastal cities.

The sample is divided into subsamples based on the values of *CoastalCity*, a variable indicating whether the firm is located in a coastal city. The results are presented Table 8. The estimated coefficients of *CorpFin×Post* are significantly positive at least at the 5% level for firms located in coastal cities. The coefficients become insignificant or relatively less significant for firms located in non-coastal cities, and the empirical *p*-values indicate that the coefficient differences between coastal and non-coastal cities are significant at the 1% level. The results suggest that following the launch of the new rules, highly financialized firms located in coastal cities are more likely to increase labor costs.

**Table 8:** Cross-sectional variation: Coastal cities.

	LaborCost_Cash		LaborCost_Total	
	Coastal City	Non-Coastal City	Coastal City	Non-Coastal City
	(1)	(2)	(3)	(4)
CorpFin×Post	23.9402***	14.2408*	23.3514**	10.9412
	(2.87)	(1.93)	(2.35)	(1.46)
Size	10.4527***	8.9029***	11.4899***	8.7531***
	(6.04)	(8.75)	(5.26)	(8.06)
Lev	-2.0155	-5.2158	-5.4299	-8.3276**
	(-0.34)	(-1.62)	(-0.81)	(-2.33)
Age	-23.1821**	-6.9227	-16.8117	-4.5909
	(-2.46)	(-1.10)	(-1.57)	(-0.69)
PPE	0.2013	0.8979	0.3235	0.8483
	(0.17)	(1.31)	(0.23)	(1.07)
OpeCashFlow	-19.9782**	-10.1638**	-11.3773	-0.3522
	(-2.38)	(-1.98)	(-1.15)	(-0.06)
ROA	-0.0458	0.0947**	-0.1124	-0.0859
	(-0.52)	(2.05)	(-1.17)	(-1.63)
SalesGrowth	0.3103***	0.3484***	0.3663***	0.4147***
	(15.83)	(28.32)	(15.75)	(27.70)
ProvGDP	2.9656	-3.6732	4.7908	-4.4499
	(0.48)	(-1.19)	(0.67)	(-1.29)
ProvGDPGrowth	-0.7411	0.1034	-0.6337	0.2497
	(-1.53)	(0.34)	(-1.02)	(0.74)
ProvPop	2.4245	7.4534	1.5087	8.6149
	(0.36)	(1.10)	(0.22)	(1.10)
ProvWage	1.3511	0.8634	6.4418	3.1891
	(0.10)	(0.12)	(0.40)	(0.39)
Constant	-220.1030	-217.4729**	-337.1565*	-248.4343**
	(-1.25)	(-2.01)	(-1.69)	(-2.03)
*Firm fixed effects*	YES	YES	YES	YES
*Year fixed effects*	YES	YES	YES	YES
*Empirical p-value*	0.006		0.007	
*N*	6565	16971	6211	16179
Adj. *R*^2^	0.367	0.396	0.354	0.391

Notes: This table investigates the heterogeneity in the baseline effects across regions. The data on labor costs, firm fundamentals, and coastal cities are obtained from the CNRDS database, and the province-level data are sourced from the CSMAR database. *CoastalCity* is an indicator variable that equals 1 if the firm is located in the coastal city, and 0 otherwise. All variables are defined in the Table 1. Robust standard errors are clustered at the firm level and *t*-statistics are reported in parentheses.

***, **, and * indicate statistical significance at the 1%, 5%, and 10% levels, respectively.

#### 4.4.3 Industry competition

Employee engagement contributes to the reduction in adverse behavior, facilitating firms’ competitiveness [18, 58]. Both financial and non-financial treatment are valued by employees [58, 59]. Research provides evidence that firms in more competitive industries tend to treat their employees more friendly in order to improve employee engagement [19]. In light of this, I expect the new rules to have a more pronounced impact on corporate labor costs for firms competing in competitive industries.

The sample is separated into high and low industry competition groups based on the values of *HighCompetition*, a variable indicating whether the firm is in an industry with above-median *Competition*. *Competition* is defined as the Herfindahl-Hirschman Index based on sales revenue. The results are shown in Table 9. The coefficients of *CorpFin×Post* are significantly positive for firms in industries with intense competition while insignificant for firms in less competitive industries, indicating that after the launch of the new rules, labor cost growth is more likely to occur when highly-financialized firms operate in industries with heavy competition.

**Table 9:** Cross-sectional variation: Industry competition.

	LaborCost_Cash		LaborCost_Total	
	More Competition	Less Competition	More Competition	Less Competition
	(1)	(2)	(3)	(4)
CorpFin×Post	24.9262**	10.0286	21.3025*	11.4687
	(2.51)	(1.26)	(1.84)	(1.25)
Size	11.0225***	9.7691***	11.3863***	9.7372***
	(7.69)	(7.66)	(6.81)	(6.97)
Lev	-3.3310	-6.5033	-7.4784	-6.8078
	(-0.80)	(-1.58)	(-1.47)	(-1.54)
Age	-25.9809***	4.4895	-24.0951***	11.1097
	(-3.65)	(0.61)	(-3.15)	(1.43)
PPE	-0.6580	1.2145	-2.2537	2.1217**
	(-0.60)	(1.43)	(-1.63)	(2.18)
OpeCashFlow	-21.4545***	-8.0554	-14.0807*	2.1559
	(-3.29)	(-1.34)	(-1.78)	(0.33)
ROA	-0.0937	0.1562***	-0.2375***	0.0009
	(-1.60)	(2.61)	(-3.35)	(0.01)
SalesGrowth	0.3627***	0.3188***	0.4344***	0.3788***
	(22.95)	(22.47)	(23.61)	(21.87)
ProvGDP	1.4558	-7.7396**	-1.1260	-9.0425**
	(0.35)	(-2.03)	(-0.24)	(-1.99)
ProvGDPGrowth	0.1763	-0.3004	0.2601	-0.0618
	(0.50)	(-0.82)	(0.62)	(-0.15)
ProvPop	9.6300	-2.6456	11.4570	-1.1003
	(1.55)	(-0.58)	(1.63)	(-0.19)
ProvWage	2.2182	-5.7970	6.6728	-3.2502
	(0.27)	(-0.72)	(0.70)	(-0.34)
Constant	-266.0003***	-68.9021	-284.8219**	-136.9944
	(-2.69)	(-0.69)	(-2.52)	(-1.12)
*Firm fixed effects*	YES	YES	YES	YES
*Year fixed effects*	YES	YES	YES	YES
*Empirical p-value*	0.011		0.094	
*N*	11831	11335	11131	10886
Adj. *R*^2^	0.418	0.367	0.408	0.369

Notes: This table investigates the heterogeneity in the baseline effects across industries. The data on labor costs and firm fundamentals are obtained from the CNRDS database, and the data on provinces and industry competition are sourced from the CSMAR database. *HighCompetition* is an indicator variable that equals 1 if the firm is in the industry with above-median Herfindahl-Hirschman Index, and 0 otherwise. All variables are defined in the Table 1. Robust standard errors are clustered at the firm level and *t*-statistics are reported in parentheses.

***, **, and * indicate statistical significance at the 1%, 5%, and 10% levels, respectively.

## 5. Further tests

### 5.1 Placebo tests

To alleviate the concern that the baseline results are driven by unobservable shocks unrelated to the new rules, I conduct a placebo test by shifting the event year by two years before the actual event year. Following Chen et al. [60], I use the variable *PseudoPost* and set the pseudo pre-treatment period as 2014–2015 and the pseudo post-treatment period as 2016–2017. Since the pseudo post-treatment period is before the publishing of the new rules, the treatment effect should be insignificant during this period. All the other variables are the same as those in the baseline regression. Table 10 presents the results. The estimated coefficients of *CorpFin×PseudoPost* are insignificant, providing evidence that the baseline results are not due to random exogenous shocks.

**Table 10:** Placebo tests.

	LaborCost_Cash	LaborCost_Total
	(1)	(2)
CorpFin×PseudoPost	7.3870	4.3269
	(1.11)	(0.56)
Size	16.1057***	18.8926***
	(9.10)	(8.83)
Lev	5.8668	-0.0440
	(1.10)	(-0.01)
Age	-40.4020**	-31.8098*
	(-2.54)	(-1.71)
PPE	1.2362	1.0179
	(1.01)	(0.75)
OpeCashFlow	-11.0693	-1.4764
	(-1.56)	(-0.18)
ROA	-0.1120	-0.4051***
	(-1.21)	(-3.57)
SalesGrowth	0.3125***	0.3691***
	(21.07)	(20.13)
ProvGDP	-14.2736*	-18.9791*
	(-1.81)	(-1.89)
ProvGDPGrowth	0.4095	0.9178
	(0.71)	(1.28)
ProvPop	11.9583	16.5251
	(1.15)	(1.29)
ProvWage	-3.8880	11.1434
	(-0.26)	(0.58)
Constant	-159.6840	-398.4904
	(-0.78)	(-1.49)
*Firm fixed effects*	YES	YES
*Year fixed effects*	YES	YES
*N*	10304	9647
Adj. *R*^2^	0.419	0.390

Notes: This table presents the results of the placebo tests. The data on labor costs and firm fundamentals are obtained from the CNRDS database, and the province-level data are sourced from the CSMAR database. *PseudoPost* is a dummy variable set equal to 1 for years 2014–2015, and 0 for years equal to or larger than 2016. All variables are defined in the Table 1. Robust standard errors are clustered at the firm level and *t*-statistics are reported in parentheses.

***, **, and * indicate statistical significance at the 1%, 5%, and 10% levels, respectively.

### 5.2 Propensity score matching

It is possible that observable heterogeneity affects the treatment effects, and therefore I re-estimate the baseline regression using a propensity-score-matched sample to address the concern. I employ one-on-one nearest neighbor matching with the caliber set to 0.01 [61] and set the dependent variable equal to one if the firm’s degree of financialization is in the upper quartile of *CorpFin*, and zero otherwise [41]. The covariate differences between the treatment and control groups are balanced using the firm- and province-level control variables specified in the baseline model. The results of the DID estimation using the matched sample is presented in Table 11. The estimated coefficients of *CorpFin×Post* are positive and significant at least at the 5% level, consistent with the baseline results. Moreover, the magnitudes of the coefficients are greater than those in the main regression, which further strengthens the baseline results.

**Table 11:** Baseline results re-estimated using a propensity-score-matched sample.

	LaborCost_Cash	LaborCost_Total
	(1)	(2)
CorpFin×Post	27.4560***	22.4402**
	(3.40)	(2.46)
Size	11.1079***	8.0909***
	(6.87)	(4.57)
Lev	-12.1822**	-18.2281***
	(-2.25)	(-3.11)
Age	-10.1414	-11.7816
	(-1.21)	(-1.16)
PPE	0.6882	2.8026**
	(0.63)	(2.04)
OpeCashFlow	-0.3983	-3.6191
	(-0.05)	(-0.37)
ROA	0.0433	-0.0145
	(0.58)	(-0.17)
SalesGrowth	0.3099***	0.3704***
	(16.99)	(16.91)
ProvGDP	-0.8979	-14.0525**
	(-0.17)	(-2.22)
ProvGDPGrowth	-0.8855*	-0.8985
	(-1.75)	(-1.40)
ProvPop	0.0657	8.3458
	(0.01)	(1.50)
ProvWage	-7.3941	12.0998
	(-0.66)	(1.05)
Constant	-117.7176	-228.9937*
	(-0.80)	(-1.67)
*Firm fixed effects*	YES	YES
*Year fixed effects*	YES	YES
*N*	8172	7894
Adj. *R*^2^	0.344	0.332

Notes: This table re-estimate the baseline model using a propensity-score-matched sample. The data on labor costs and firm fundamentals are obtained from the CNRDS database, and the province-level data are sourced from the CSMAR database. All variables are defined in the Table 1. Robust standard errors are clustered at the firm level and *t*-statistics are reported in parentheses.

***, **, and * indicate statistical significance at the 1%, 5%, and 10% levels, respectively.

### 5.3 Other robustness tests

The new rules are officially published in April 2018, and *CorpFin* in the baseline model measures ex ante degrees of corporate financialization using data one year prior to the new rules. Here, I adopt alternative measures by expanding the time horizon for calculating firms’ ex ante degrees of financialization. Specifically, *CorpFin_2yr* is defined as the average ratio of financial assets to total assets using data two year prior to the new rules, and *CorpFin_PreTreat* is based on the data during the pre-treatment period. I then replace *CorpFin* with *CorpFin_2yr* and *CorpFin_PreTreat*, respectively, and re-estimate Eq (1). Panel A of Table 12 reports the results. The coefficients of the variable of interest are significantly positive across the columns, suggesting that the baseline results are robust to alternative measures of corporate financialization with different time horizons.

**Table 12:** Robustness tests: Alternative specifications.

**Panel A: Alternative measure of firms’ degree of financialization**				
	LaborCost_Cash	LaborCost_Total	LaborCost_Cash	LaborCost_Total
	(1)	(2)	(3)	(4)
CorpFin_2Yr×Post	17.0319***	14.2842**		
	(2.90)	(2.19)		
CorpFin_PreTreat×Post			15.8673**	12.7518*
			(2.56)	(1.77)
*Controls*	YES	YES	YES	YES
*Firm fixed effects*	YES	YES	YES	YES
*Year fixed effects*	YES	YES	YES	YES
*N*	21843	21133	19498	19231
Adj. *R*^2^	0.384	0.376	0.375	0.368
**Panel B: Alternative sets of fixed effects**				
	LaborCost_Cash		LaborCost_Total	
	(1)		(2)	
CorpFin×Post	16.1297***		14.0839**	
	(2.97)		(2.34)	
*Controls*	YES		YES	
*Firm fixed effects*	YES		YES	
*Year fixed effects*	YES		YES	
*Province fixed effects*	YES		YES	
*N*	23552		22406	
Adj. *R*^2^	0.388		0.380	
**Panel C: Alternative samples**				
	LaborCost_Cash		LaborCost_Total	
	(1)		(2)	
CorpFin×Post	14.1033**		13.2632**	
	(2.41)		(2.06)	
*Controls*	YES		YES	
*Firm fixed effects*	YES		YES	
*Year fixed effects*	YES		YES	
*N*	22590		21450	
Adj. *R*^2^	0.405		0.399	

Notes: This table conducts robustness tests using alternative specifications. The data on labor costs and firm fundamentals are obtained from the CNRDS database, and the province-level data are sourced from the CSMAR database. *CorpFin_2Yr* and *CorpFin_PreTreat* are the average ratios of the firm’s financial assets to its total assets two years prior to and during the pre-treatment period of the new rules’ launch, respectively. All variables are defined in the Table 1. Robust standard errors are clustered at the firm level and *t*-statistics are reported in parentheses.

***, **, and * indicate statistical significance at the 1%, 5%, and 10% levels, respectively.

Since labor markets are partially shaped by regional characteristics, I add province fixed effects into Eq (1) and re-estimate the impact of the new rules on corporate employment decisions. The results are shown in Panel B of Table 12. The coefficients of *CorpFin×Post* remain significantly positive at the 5% level to alternative fixed effects, providing evidence that the baseline effects are robust to different specifications.

Following the literature [31], financial firms are excluded from the full sample in the main analyses. In light of the fact that firms in the real estate and insurance industries have gone further in corporate financialization, I exclude firms in these two industries and re-estimate Eq (1). Panel C of Table 12 reports the results. The coefficients of *CorpFin×Post* are positive and significant at the 5% level, suggesting that the baseline results are not largely driven by firms in the real estate and insurance industries and that firms in other non-financial sectors do indeed respond to the new rules by increasing labor costs.

### 5.4 Additional tests

After the launch of the new rules, highly financialized firms increase labor costs, providing evidence for their redirection towards the real economy. These firms’ labor cost growth may be partly attributed to an increase in employment size. I directly investigate whether firms more exposed to the new rules recruit more people by using *LaborN* as the dependent variable in Eq (1). *Labor_N* is defined as the change in the firm’s number of rank-and-file employees. The results are presented in Table 13. The coefficient of *CorpFin×Post* is positive and significant at the 1% level, showing that after the publishing of the new rules, firms with larger exposure to the new rules recruit more people. Combined with this additional finding, the results provide evidence that firms with ex ante higher degrees of financialization redirect to real-economy activities by increasing wages and employment size.

**Table 13:** Additional analyses.

	LaborN
	(1)
CorpFin×Post	452.3584***
	(2.66)
*Controls*	YES
*Firm fixed effects*	YES
*Year fixed effects*	YES
*N*	23504
Adj. *R*^2^	0.258

Notes: This table investigate the effect of financialization disruption on employee size. The data on firm fundamentals are obtained from the CNRDS database, and the data on provinces and labor size are sourced from the CSMAR database. *LaborN* is the change in the number of rank-and-file employees. All variables are defined in the Table 1. Robust standard errors are clustered at the firm level and *t*-statistics are reported in parentheses.

***, **, and * indicate statistical significance at the 1%, 5%, and 10% levels, respectively.

## 6. Discussion

Broadly, the paper contributes to the literature on regulatory arbitrage by showing that a unified regulatory framework could have unintended labor market consequences. China’s financial industry regulators have begun introducing the unified framework, in terms of policy design and organizational structure, to the regulatory system, which combats regulatory arbitrage arising from overlapping responsibilities. There is, however, a scarcity of empirical research that examines the bright side of unified financial regulation [1]. This study fills the void by investigating the externality of unified financial regulation and its interaction with corporate agency costs. The findings demonstrate that curbing regulatory arbitrage disrupts corporate financialization and prompts non-financial firms to refocus on the real economy and improve corporate governance, extending the understanding that unified regulation could not only promote economic development but also benefit social welfare.

Furthermore, this study makes contributions to the line of research on corporate de-financialization in emerging markets. Prior studies focus on the motivations and consequences of corporate financialization, and a strand of literature has evidenced that financial investments would crowd out real investments [3–6]. Nevertheless, few studies investigate the effects of corporate de-financialization. This paper exploits the exogenous shock that disrupts firms’ process of financialization and finds that microeconomic agents in the emerging market, Chinese firms in the real sector, make efforts in de-financialization by increasing labor costs as a response to changes in financial regulation.

The research is subject to limitations. The empirical analysis is based on listed firms and might not generalize to non-listed firms. The effects of the unified new rules on corporate employment strategies pertain to China and might not generalize to economies with different institutional environments.

## 7. Conclusion

In order to control systemic financial risk and improve financial stability, China’s new asset management rules, rolled out in April 2018, bring financial institutions under the unified regulatory framework in an attempt to reduce regulatory arbitrage. Extant literature mainly focuses on financial institutions’ regulatory arbitrage and non-financial firms’ motivation for and capital market consequences of financialization. Little is known about how the regulation-induced disruption of financialization shapes corporate employment decisions and imposes real effects on the labor market. This study addresses the research gap.

Using a sample of Chinese listed firms during 2014–2021, I examine how the exogenous shock to corporate financialization affect firms’ labor costs. The baseline results show that firms with ex ante higher degrees of financialization respond to the new rules by larger increases in labor costs. Moreover, I investigate the agency cost mechanisms through which financialization disruption affects corporate employment decisions and find that highly financialized firms are more likely to increase labor costs when they have less financially savvy management and lower ownership concentration. Furthermore, heterogeneity across firms, regions, and industries exists in the impact of the new rules on corporate labor costs. Specifically, after the launch of the new rules, firms with ex ante higher degrees of financialization are more likely to experience labor cost growth when exposed to lower bankruptcy risk, located in coastal cities, and operating in industries with heavy competition.

The results yield multiple implications for policymakers. First, given China’s institutional environment, the unified regulatory framework is critical in designing policies that prevent financial instability and improve regulation efficiency. Second, with the objective to identify potential financial risks, regulatory authorities should be equipped with state-of-the-art technology in distinguishing financial innovations that facilitate corporate financialization, especially in the presence of financially savvy management. Third, in order to exert a profound effect on the real economy, the new asset management rules should be accompanied by policies on expanding corporate financing and improving corporate governance.

The study suggests several avenues for future research. First, granular data on corporate employment flows could provide a deeper analysis on the effect of the new rules. Another research direction is to examine the impacts of the new rules on other important corporate decisions, for example, ESG strategies. Moreover, future research could explore how other dimensions of the unified regulatory framework, including adjustments to organizational structure, exert influence on corporate employment decisions.
